# Parent and implementer attitudes on gender-equal caregiving in theory and practice: perspectives on the impact of a community-led parenting empowerment program in rural Kenya and Zambia

**DOI:** 10.1186/s40359-022-00866-w

**Published:** 2022-06-27

**Authors:** Kenneth Okelo, Silas Onyango, Dawn Murdock, Kaylie Cordingley, Kelvin Munsongo, George Nyamor, Patricia Kitsao-Wekulo

**Affiliations:** 1 African Population and Health Research Center, Nairobi, Kenya; 2 Episcopal Relief & Development, New York, USA; 3 Zambia Anglican Council Outreach Programmes (ZACOP), Lusaka, Zambia; 4 Anglican Church of Kenya Development Services-Nyanza (ADS-Nyanza), Kisumu, Kenya

**Keywords:** Fathers’ roles, Fathers’ perceptions, Fathers’ practices, Responsive care, Childcare, Stimulation, Early learning, And holistic development, Early childhood development, Parenting

## Abstract

**Background:**

Fathers are often perceived to be mainly responsible for the provision of the family's economic needs. However, past studies have demonstrated that fathers’ involvement in parenting has great significance for the child’s holistic growth and development. Few studies have investigated fathers’ roles in the nurturing care of young children, particularly responsive care and stimulation, in sub-Saharan Africa. The study reported here was carried out as part of a larger study that sought to evaluate the effectiveness of the *Moments That Matter* (MTM) program in improving the nurturing care of young children in rural communities in Zambia and Kenya. The MTM program uses a parenting empowerment approach to promote bonding and interactions between caregivers and their children within the home, focusing on responsive care, early learning, and security and safety so that children reach their full developmental potential. Trained volunteers facilitated monthly primary caregiver support and learning groups and ECD home visits. Fathers were encouraged to participate in the home visits and to attend some of the group meetings on specific topics. The study reported in this paper aimed to establish the impact of the parenting empowerment program in promoting more gender-equal attitudes and practices on parenting among fathers (who were not the primary caregivers).

**Methods:**

Qualitative data were collected at three time points (pre-intervention before the implementation began; mid-intervention after 6 months of implementation; and post-intervention, after 24 months). We conducted focused group discussions with primary caregivers (n = 72) and fathers (n = 24) with children below 3 years. In-depth interviews were conducted with ECD Promoters (n = 43) and faith leaders (= 20). We also conducted key informant interviews with the MTM program implementers (n = 8) and government officials (n = 5) involved in the program implementation. We employed thematic analysis to analyse the qualitative data.

**Results:**

The findings showed that the MTM program resulted in improved gender-equal parenting attitudes and practices among mothers/other primary caregivers and fathers. Study participants reported that most fathers spent more time playing and interacting with their children and were more involved in household chores due to their participation in the MTM program.

**Conclusion:**

The study findings provide evidence for policy formulation and a guide for implementation of policies that can influence changes in perceived gender roles in parenting.

## Background

In many communities particularly within sub-Saharan Africa (SSA), fathers play an important role in decision-making within the home, providing financial support and are traditionally considered the main breadwinners [1]. As pointed out by Erzse and her collegues, in SSA, both men and elderly women support patriarchal gender divisions of labour, which means that women are primarily responsible for early life nutrition and care [2]. A mother’s responsibility in parenting traditionally included all “activities within the home, such as feeding, cooking, bathing and cleaning the house” [p. 21] [3]. Their role in parenting is perceived as an authoritarian discipliner and their relationship with their children is often characterized by fear [4]. These roles and responsibilities based on a person's gender are reinforced during traditional lessons that a couple undergoes before marriage. Many fathers still believe that caring for a child is mainly the responsibility of the mother, especially during the first one to two years of a child's life [2].

A father figure in the child’s environment is more likely to help the child learn that caring is part of masculinity as well as femininity [5]. Moreover, both parents’ involvement in childcare has significant benefits for children’s development [6], survival and health. For instance, father-child interaction has been shown to improve children’s cognitive, social, language and emotional development [7]. Other examples of positive long-term effects of father involvement include better social functioning during childhood, higher educational attainment and lower incidences of delinquency and criminal behaviour [8]. Further, the involvement of fathers early on in a child’s life results in the father’s satisfaction which in turn leads to a greater likelihood of sustained involvement as the child grows older [9].

Interactions between the father and the child may mirror representations and recollections of the father’s own childhood experiences [10]. Beliefs about gender roles have also been cited as a major factor hindering fathers’ participation in childcare [11]. Other barriers that have been identified include social-cultural issues, limited maternal health knowledge, and stigmatization of fathers who participate in roles attributed to females, health structures and poor information on the importance of fathers’ involvement [12]. Such barriers can be addressed through parenting empowerment programs.

Gender-responsive parenting initiatives have shown potential for positive gender norm change in the recent past [13]. Implementation of such gender-responsive programs with a strong emphasis on the engagement of male caregivers have yielded positive outcomes. Four initiatives by UNICEF in Nepal, Sri Lanka, Ghana, and Tanzania reported improved father involvement in childcare [13]. Such initiatives integrated stronger gender equity focusing into ongoing work in early childhood development (ECD) promoting good nutrition. Despite the importance of such gender-responsive parental programs in changing gender norms, few programs have been implemented in countries in SSA such as Kenya and Zambia.

**Fig. 1:**
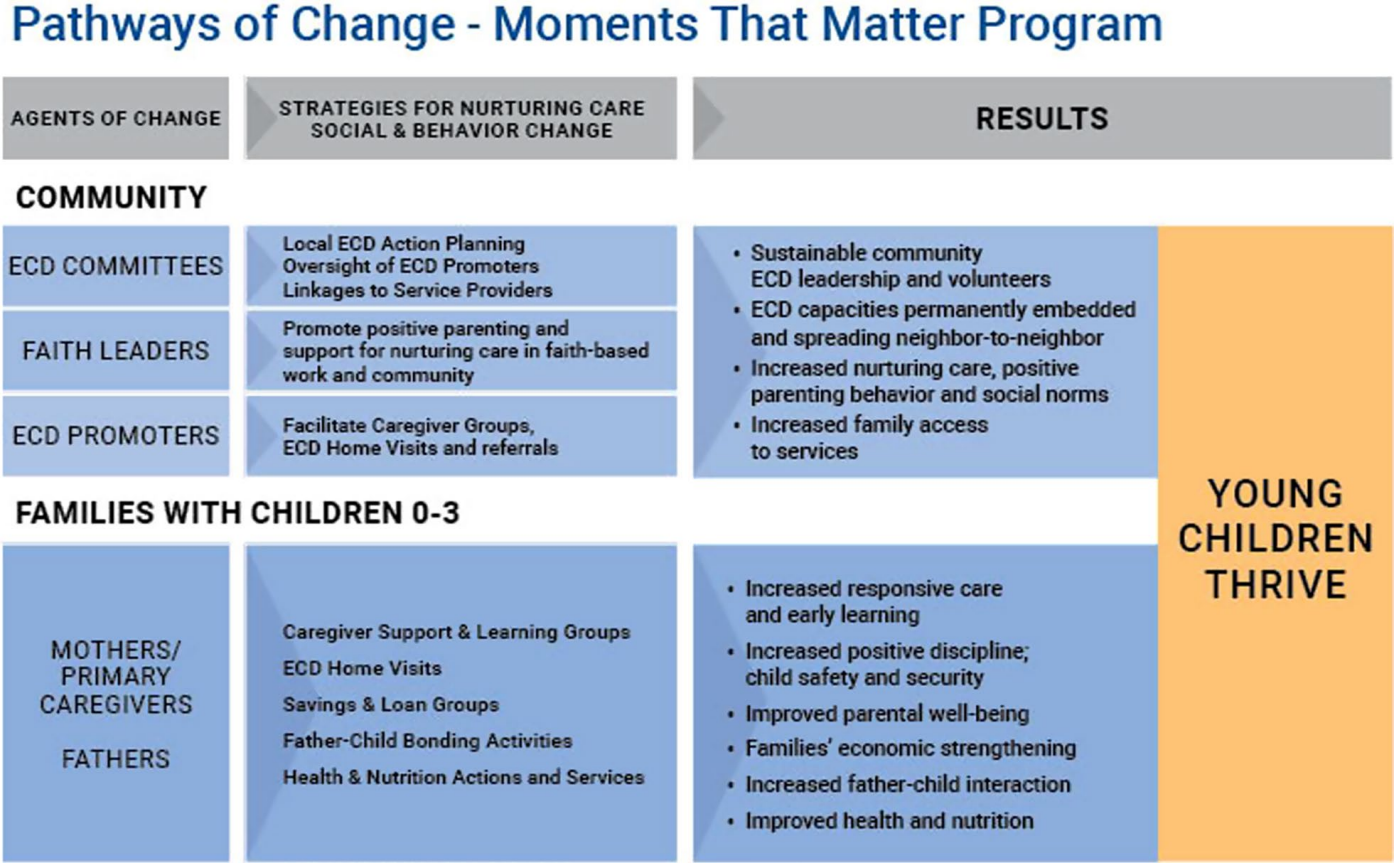
Pathways of change of the *Moments That Matter* Program

The *Moments That Matter* (MTM) program, a partnership of Episcopal Relief & Development and Anglican Development Services of Nyanza (ADS-Nyanza) in Kenya, and Zambia Anglican Council Outreach Programmes (ZACOP) in Zambia, takes a parenting empowerment approach to integrated early childhood development from birth to three years. The MTM program mobilizes rural communities for nurturing care and parenting empowerment, through a holistic, community-led approach engaging the most vulnerable families with a pregnancy and/or children aged zero to three years [14]. Vulnerabilities included: HIV-affected, other chronic or severe illnesses, single parent households, grandparents or adolescent caregivers, parent or child with disabilities, and food-insecurity. The marginalized rural communities are characterized by high poverty rates, smallholder farming, poor maternal and child health and nutrition, high HIV prevalence, long distances to health services, and limited access to financial services. The MTM program promotes parental empowerment, bonding and interactions in the home between caregivers and their children, focusing on responsive care, early learning, and security and safety so that children reach their full developmental potential. Trained ECD volunteers facilitated monthly Caregiver Support and Learning Groups (CS&LG) combined with ECD home visits. The MTM program also aimed at improving fathers’ participation in caregiving practices and intentionally included activities and messages around gender-equitable parenting between male and female caregivers (encouraging male participation in caregiving) and providing gender-equitable care for boys and girls. In addition, MTM training of faith leaders and the use of male ECD volunteers also reinforced male caregivers' involvement in parenting (See Fig. 1). The study reported in this paper aimed to establish the effects of the parenting empowerment program on more gender-equal attitudes about gender roles in parenting and actual caregiving practices by fathers (who were not the primary caregivers), after participation in the MTM program in Kenya and Zambia.

We explored the type of attitudes and practices that existed before the program implementation (baseline) and pointed out any changes that may have occurred during the program implementation (midline) and after two years of the implementation period (endline).

## Methods

### Study design

The qualitative study reported in the current paper was part of a quasi-experimental study in which caregiver-child dyads were assigned to the intervention (to receive the MTM program intervention) and the control arm (to receive the standard care, i.e., routine child health monitoring delivered by community health volunteers) [14]. The villages or clusters were purposively selected, taking into consideration such factors as poverty levels and the number of families, to make them as comparable to each other as possible with regards to their demographic characteristics.

*Kenya* Three clusters from each of the two sub-locations (Ayucha and Border 1) within the program implementation sites and six clusters from a third sub-location (Wangang’a) where there were no program activities were selected.

*Zambia* Ten communities serving as the clusters were formed by grouping villages within the Chamuka area program site. Within the study area, villages were randomly selected from program sites and stratified at the cluster/community level to reduce potential contamination among study arms. Three to four villages (depending on the number of households in each village and geographical location) were clustered into five “community” program implementation sites from one ward (Mwantaya) and allocated to the intervention arm, and five communities located in a different ward (Chamuka) were allocated to the control arm [14].

### Sampling and sampling characteristics

In the main study in Kenya, 244 primary caregivers were recruited at baseline and 121 were followed up at endline surveys (32.3% attrition rate) in the intervention and control arms. The research team also recruited more (N = 44) study participants from the intervention arm who had been participating in the MTM program activities bringing the number of study participants in the intervention arm at endline to 165. In Zambia, the research team recruited 395 primary caregivers at baseline, with children aged below 18 months or were pregnant and were in their third trimester) were identified and recruited and 194 followed up at endline. The attrition rate was 43.9% (n = 92) in the intervention arm and 52.8% (n = 109) in the control arm. In both countries, slightly above three-quarters of the respondents mentioned that they had a father present in their household [14].

In the study reported in this paper, the research team used two types of purposeful sampling strategies for qualitative data collection. Purposeful sampling enabled us to identify and select information-rich cases for the phenomena under study. The theoretical assumption underlying the sampling of participants for the qualitative interviews is the attainment of the data saturation point, that is, the point at which no new substantive information is obtained from additional interviews [15]. The selection of respondents for the qualitative interviews was based on (a) maximum variation sampling to document or identify unique or diverse variations within a homogenous population, and to enable the identification of common patterns that emerge; and, (b) the selection of homogenous cases to facilitate group interviewing [16]. Whereas maximum variation sampling allowed us to identify differences among the respondents, homogenous sampling focused on identifying similarities among those who were interviewed (Tables 1 and 2). We purposely selected program volunteers who were directly involved in the implementation of the MTM program activities for the key informant and in-depth interviews. We conducted structured qualitative interviews with the respondents.

**Table 1:** Tools used during each phase of data collection

	Kenya			Zambia		
Interview type	Pre-implementation	Mid-intervention	Post-intervention	Pre-implementation	Mid-intervention	Post-intervention
Focused group discussions	13	12	16	15	9	9
In-depth interviews	15	15	17	18	17	47
Key informant interviews	6	9	8	3	2	2

**Table 2:** Participant characteristics

Interview type	Participant category	Gender		Marital status		Education level		Main occupation			Mean age
		Male	Female	Not married	Married	Primary and below	Post primary	Employed	Self-employed	Unemployed	
In-depth interviews	ECD promoter	8	19	3	24	7	20	3	28	0	33
	Lead ECD promoter	6	10	2	14	2	14	2	8	0	30
	ECD committee: chair, other officials, member	14	7	0	21	0	21	15	6	0	42
Focused group discussions	Primary caregivers	0	72	10.8	61.2	54	18	8	40	24	32
	Fathers	24	0	0	24	12	12	8	7	8	38
	MTM-trained faith leaders	14	6	0	20	5	15	11	9	0	35
	CHVs	12	31	2	41	32	11	0	0	0	39
Key informant interviews	Program staff	2	6	1	7	0	8	8	0	0	40
	Policy implementers	2	3	0	5	0	5	5	0	0	42

### Study setting

The study was conducted in two countries, Kenya and Zambia among populations with similar poverty levels (Kenya; 32.5% and Zambia; 37%) and demographic characteristics.

*Kenya* The study was conducted in Border 1 and Ayucha sub-locations in Awasi-Onjiko Ward within Nyando sub-County in Kisumu County. Kisumu County in the Nyanza region is among the 47 semi-autonomous county governments, which were formed after the promulgation of the Kenyan constitution in 2010. Every county is further subdivided into sub-counties and wards for ease of administration [17, 18]. Kisumu County has an overall poverty rate of 32.5% [19]. The county has six administrative sub-counties (Kisumu West, Kisumu East, Kisumu Central, Seme, Nyando, Nyakach, and Muhoroni). Nyando sub-County has five wards (Awasi-Onjiko, Ahero, Kabonyo/Kanyagwal, Kobura, and East Kano/Wawidhi) with a total population of 141, 037. Males account for about 51% of the total population. The infant mortality rate is 24 deaths/1000 live births [19].

*Zambia* The study was conducted in the Chamuka area of Chisamba District of Central Province. Central Province has an overall poverty rate of 37%. The level of rural poverty is three times that of urban areas. The Chamuka area has over 30 villages in two wards, Mwantaya and Chamuka Wards (note: Chamuka Ward has the same name as the larger area). The infant mortality rate is 42 deaths/1000 live births [14].

### Data collection procedure

The researchers conducted qualitative interviews [focus group discussions (FGDs) and in-depth interviews (IDIs)] with fathers, primary caregivers, community volunteers, The MTM program implementers and government policy implementers to establish their perceptions and practices on gender roles in parenting. Trained field interviewers who had a graduate qualification and experience in conducting qualitative interviews conducted the interviews. The field interviewers were trained for 5 days by the research team. During the training which involved theoretical sessions, role plays and a field pilot to ensure that the field interviewers were conversant with the interview guides, we emphasized the need for them to engage in a reflexive approach and strive to be as neutral as possible during the data collection activities. In this way, we were able to reduce bias and minimize judgmental behaviours. The interview guides were further refined based on the feedback from the pilot exercise.

Each interview was managed by a pair of field interviewers i.e., a moderator who led the discussion and an assistant who took notes from the discussions. The FGDs with caregivers, community volunteers, religious leaders and Community Health Workers (CHWs) were conducted in the most commonly used language used in these communities (Dholuo in Kenya and Tonga and Nyanja in Zambia). Key informant interviews (KIIs) with sub-county/District officials and program staff were conducted in English. We interviewed program staff to understand how the MTM program has been implemented and their perceived behaviour change among parents on gender role in caregiving. In addition to taking notes, all the discussions and interviews were audio-recorded with the consent of the participants to maximize the completeness of the information collected and reduce the risk of data loss. The field supervisor reviewed the notes and the audio files to ensure that all the items in the interview guide were comprehensively covered. The length of FGDs varied from one to one-and-a-half hours while the KIIs were between 45 min and 1 h.

### Analysis

The researchers employed thematic analysis. The audio-recorded files from the interviews were transcribed in English and anonymized by a qualified and experienced transcriber. In order to ensure that the transcripts were culturally neutral, the transcriber excluded colloquial language, and corrected all grammatical and typographical errors. After the transcription, the research team checked each transcript for accuracy and quality assurance thereby ensuring that the transcripts represented the information in the audio-recorded files. The research team including the principal investigator, a qualitative researcher and three junior researchers then used the interview guides and initial transcripts to identify the main themes and sub-themes. This informed the development of the codebook which was used to code the remaining transcripts. All the coded data were then classified and analysed through word trees and queries in Nvivo Q 10 Software using thematic content analysis [20]. To ensure the trustworthiness of the thematic analysis, and that the process was credible, transferable, dependable and confirmable, the research team developed an analysis plan [21]. The analysis plan was used as a point of reference during transcription, coding and analysis. In addition, the research team recognized that their positionality changed over the course of the three data collection time points. When the data collection process began, we were considered as outsiders. However, as more contact and discussion with the research participants took place, they increasingly viewed us as being insiders due to familiarity during the follow-up period. The research team held a data interpretation workshop to ensure that there was a common understanding of the emerging themes, and that any biases in the analysis process were minimized.

## Results

We present information on community members’ perceptions and practices on equitable gender roles and fathers’ involvement in caregiving after participating in the MTM program for two years. This includes changes in attitude, knowledge levels and parenting practices.

### Attitudes towards gender equality in parenting

At baseline, most respondents reported that childcare was the sole responsibility of mothers or the primary caregivers. Fathers were reported to be undertaking childcare responsibilities when mothers were unwell or not at home. Fathers and mothers narrated that the separation of parenting responsibilities stemmed from how they were brought up. Post-intervention at endline, there was evidence of changes in parenting attitudes and practices towards more gender equity.

*“They [fathers] provide money to buy food for the children if you don’t have or as a mother you can buy for them some clothes so that they have a better life” FGD with primary caregivers, Zambia at baseline*

*“Children in the past, just like it was when we were growing up, weren’t free around their fathers. Fathers would chase the child asking the mother to get their child as though the mother was the only person responsible for birthing the child. Today’s children who were born into this program have no reason to fear their parents. Children and other parents from back in our time get surprised that fathers are taking care of children, playing with them. They say we have spoiled them.”* FGD with primary caregivers, intervention, Zambia at endline

*“In the past, the way we were raised, my father or my elder brother were like lions, whenever they entered a house, I would always look for a door in order to try and escape, but these days they teach is to be friendly with the children, these days’ children are not afraid of us, we also hug the children.”* FGD with fathers, intervention, Zambia at endline

This notion changed at midline and further changes were noted at endline. At the endline, in both countries, some primary caregivers mentioned in the qualitative interviews that fathers had embraced the notion that they could also provide caregiving. Primary caregivers added that fathers were more involved than before; they had become close to their children, were involved in play with their children and supported their wives during pregnancy and with different household activities such as cleaning. Through fathers’ participation in the program activities, their parenting skills seemed to have improved.

*“After sharing the knowledge we received through the MTM program with our husbands, they are now involved in caring for their families. They feed their children, take them to the clinic, and support wives during pregnancy. Previously, fathers used to think that their responsibility was only to provide materially for their families. Nevertheless, after the training, they changed their attitude. They now play with children and care for pregnant mothers. For instance, he postpones other activities to care for pregnant mothers and provides a balanced diet too.” FGD with female primary caregivers, intervention, Kenya at endline*

*“Male caregivers are involved more in caring for their children. Some take time to stroll with their children as their wives care for other activities. Furthermore, fathers take their children to school both in the morning and evening besides making play items for them.” IDI with ECD Promoter, Kenya at endline*

*We also fathers now playing with their children. They carry their children around; fathers also wash and change diapers for their children. Children love their parents and they are very close. In fact, they are closer to their fathers than mothers because of the training this program offered to fathers. IDI with ECD Promoter, Zambia at endline*

Notably, in the control arm, some fathers also mentioned attitudinal changes towards caregiving. However, their sentiments were centered only on the provision of basic needs and psychological support to the mother during pregnancy. It was evident in the control arms that many caregivers still held the opinion that caregiving was the sole responsibility of mothers for children below two years.

“The work of a mother in parenting children is to take full care of the children, cooking good food like porridge for babies, washing their clothes, taking the child to the nearest health post for under-five.” FGD with fathers, control, Zambia at endline

“The woman is the one who spends the most time with children, we men can have things like work, but if women spend too much time away from the child, then the child will not breastfeed well, and their health will go down. If the child is one year and six months old and is left with fellow children with no proper food, the children will not take care of that child the way the mother would. We men can be close to the child even from birth, but we only have a hand in the child’s life when they are old enough to be away from the mother when we can carry the child to the market or something of that sort.” FGD with fathers, control, Zambia at endline Parenting behaviour change toward gender equal roles.

In both countries, before the implementation of the MTM program, parenting responsibilities were delineated according to gender. Most mothers were entirely responsible for their children. Activities such as bathing, feeding, dressing, healthcare, and spending time with children were perceived to be a sole mandate of mothers. Fathers were expected to provide enabling means to mothers. However, both mothers and fathers provided information during qualitative interviews on how the MTM program has influenced gender roles in not only parenting but at the household level in general.

The participants from the intervention arm mentioned that fathers were more involved in household chores. The respondents attributed these changes to the MTM program activities. ECD Promoters also reported that through the mentorship they provided during household visits and caregiver group meetings, male caregivers had become more positive about participating in childcare and household chores.

*“After the ECD training, both male and female caregivers participated in childcare and doing other domestic chores…. They are responding well. We can now see fathers cooking for their expectant wives besides going to the facility for clinics. Even this morning, I saw a father carrying a child… The fathers are helping their wives with some chores at home. When we are conducting our routine household visits, they warmly welcome us even in the absence of their wives.”* IDI with Lead ECD Promoter, Kenya at endline

*“In the past, I used to leave all the house chores in the hands of my wife. She was doing everything. Nowadays I help her with the chores in the house… I hold the baby, do the washing and cooking.”* IDI with a father, intervention, Kenya at endline

*“You’ll find that you’re tired from working in the field and so is your husband, but they will still want you to get home and do all the chores including taking care of the child. So, we have been taught that fathers are also supposed to take up a supportive role in the upbringing of children.”* FGD with female primary caregivers, intervention, Zambia at endline

*“I never used to play with my child or clean him when he poops, and when the child does something wrong, I was quick to get a whip to beat the child but I don’t do all these things.”* FGD with fathers, intervention, Zambia at endline

*“The program has taught us a lot, especially regarding gender. We never knew that men could also have a role in the upbringing of their children. Even if you went far and you come back, you will find your husband has cooked, fed the children, and even put water for you to bathe. We would in the old days do all these tasks, but that’s not the case today.”* FGD with female primary caregivers, intervention, Zambia at endline

The MTM program positively influenced sharing of household and parenting responsibilities among mothers and fathers. Fathers had become closer to, increased the time spent with their children, and took up more responsibilities. Thus, fathers´ emotional connections with their children improved considerably, which in turn provided a more enabling environment for children to thrive. In addition to an improved relationship between fathers and children, the enhanced participation of fathers in daily household routines also resulted in overall improved relationship quality among spouses.

*“Through what we learnt I have seen that it is good for us to help each other, therefore, the only job that I know is strictly for the woman is giving birth, but the rest we can help each other. All of us can help, washing especially should be for men, for example, when the woman is pregnant and about to deliver... they may keep her for some days at the clinic. When she is away, I have to bathe my children. What am I going to lose by bathing my children? So everything we need to do is to help each other.”* FGD with fathers, intervention, Zambia at endline

*“Yes, there has been an improvement in my relationship with my wife. In the past, she was not happy, because she could not express herself properly to me. But now that I have learnt, she is more open to me, so there is an improvement in the way we relate, she has the freedom and can count on me for help.”* FGD with fathers, intervention, Zambia at endline

*“It is very true, I never had time to listen to a woman and her opinion, when she was giving me advice, I just thought she was wasting my time, but through the lessons, I have learnt to value her opinion, these days when she says something we sit together to discuss what she thinks.”* FGD with fathers, intervention, Zambia at endline

### Uptake of maternal and child health (MCH) services

In both countries, before the MTM program, seeking healthcare for children was usually seen as the mothers´ responsibility. This meant that mothers had to walk long distances with their children tied on their backs to attend the monthly under-five clinic or seek healthcare when the child felt unwell. Most fathers did not participate in this task and many did not show interest in a child's health/physical development. At endline, the qualitative interviews revealed that the participants perceived that the MTM program had improved male caregivers’ participation in the MCH services such as antenatal care (ANC) services, nutrition and immunization, and preventive services including attending sessions on proper handwashing. They reported that more male caregivers accompanied their wives to the hospital.

*“In the past, my husband never bothered with my antenatal visits. After the training, he now inquires about my clinical visits and even accompanies me to the hospital. At home, he discourages me from stressful duties and advises me. This makes my work easier… Previously, my husband believed that taking children to the hospital was the role of the mother. Recently, when I was sick, he took the child to the clinic when the child became ill.”* FGD with female primary caregivers, intervention, Kenya at endline

*“Fathers are now taking children to clinics and are involved in processing birth certificates.”* IDI with Lead ECD Promoter, Kenya at endline

*“The benefit I found is that long ago things were hard for women because here the health centre is very far, but now the fathers help in taking care of the children even taking them to the clinic, but in the past, he would just leave everything to me to carry the child to the clinic.”* FGD with female primary caregivers, intervention, Zambia at endline

*“Me on my part, the benefits I have seen in my life are a lot, for example when my wife is going to the clinic for under five, I will get the child and put it on my back and then she will only get her handbag. I even see her get happy.”* FGD with fathers, intervention, Zambia at endline

*“There is change because of the teaching they give us. We see fathers carrying their children on the back and going to the clinics that never used to happen. They even bathe their children, even in our community we have seen the change. We see fathers feeding their children and doing things that they never used to do.”* FGD with fathers, intervention, Zambia at endline

It is somewhat remarkable that fathers engaged in what is traditionally seen as the responsibility of a woman in the community despite resistance and mockery from other fathers in the community. Fathers narrated that men who helped their wives in chores that were perceived to be a woman´s responsibility were considered fools, weak, or bewitched.

*“The other issue was escorting the wife to the clinic when our child was sick. Other men used to laugh at me, but these days I just consider them as fools and ignore them, even if they laugh, because I know the benefits of helping and taking care of my child. I just get my bicycle and take the child to the clinic, I don’t hesitate.”* FGD with fathers, intervention, Zambia at endline

*“Helping my wife carry firewood, if I help her carry the heavy ones, they will say she has finished me as a man, and I have just become a fool.”* FGD with fathers, intervention, Zambia at endline

*The community people used to laugh at me for example, when I carry my baby on my back, the moment I leave home people will start laughing at me, they will say I am a fool and my wife has given me charms.”* FGD with fathers, intervention, Zambia at endline

## Discussion

This study aimed to examine whether gender roles and fathers’ attitudes, knowledge, and childcare practices changed as a result of participation in the MTM program. From the findings, participants gained knowledge about gender roles in parenting which was demonstrated through male caregivers’ involvement in childcare responsibilities. The current study showed an attitude and behaviour change on parenting responsibilities, especially with regards to responsive caregiving and stimulation practices. Earlier evidence shows that spending time with the child leads to improvement in language and social skills [22]. Studies have also shown the importance of a father’s involvement in childcare responsibilities in improving children’s developmental outcomes and women’s economic empowerment [5]. Therefore, their increased involvement in caregiving activities has the potential of improving child development outcomes.

Notably, some participants still held the opinion that due to the nature of the father’s work in the rural communities, they might not find adequate time to engage their children in play and stimulation activities. A similar finding showed limited participation in caregiving activities by fathers, which have been associated with the fathers’ childhood experiences [10]. In most rural communities in Africa, parenting roles are delineated based on gender [4]. This separation of parenting roles along gender lines often resulted in a complete separation between fathers and their children [4]. Such gender norms which dictate responsibilities inside and outside the households are often more evident in African rural communities. Evidence from other studies shows that fathers have unique roles in childcare that may differ from those of mothers as demonstrated in this study; some are actively involved in caregiving roles. Findings from other studies also indicate the importance of fathers’ engagement on children’s cognitive, social, language and emotional development.

Widely held societal beliefs that women are responsible for childcare inform women being the main beneficiaries and receivers of information and programs involving children. In addition, childcare policies emphasize mothers, especially women of reproductive age, pregnant and lactating mothers. Such policies on breastfeeding, maternal and child health, maternity leave have led to development programs that support women in caregiving activities [23]. These programs have improved child development and the mother’s health outcomes. However, there are few policies on fathers’ participation in childcare. While women are the primary focus in stimulating children's development outcomes in the first three years of the child's life, in some communities they have limited decision-making power and resource allocation [24]. Therefore, policies and programs need to focus on empowering both parents to promote the holistic growth and development of their children.

### Study limitations

One of the limitations of this study is that since the data were self-reported, there was the risk that fathers exaggerated their responses to increase social desirability, or under-report on those aspects that they considered being problematic. However, this was mitigated by qualitative interviews with other respondents such as primary caregivers, program implementers, religious leaders and policy implementers who reported fathers’ attitudes and behaviour changes on gender roles in parenting.

## Conclusions

Perception of more equitable gender roles in parenting and fathers’ participation in childcare improved among the program participants with most of them reporting that fathers were actively involved in parenting and caregiving activities due to their participation in the MTM program. Those who reported low participation in parenting/childcare pointed out gender stereotypes as major barriers to their participation. Therefore, a community-based parenting empowerment program could be instrumental in addressing such stereotypes as demonstrated in the current study.

### Implications

Given that gender stereotypes embedded in traditional beliefs on parenting roles may influence fathers’ engagement with young children, a community-led parenting empowerment program such as the one reported in the current paper could be considered instrumental in addressing this situation. Increased father involvement is important as it impacts children’s short-term and long-term outcomes. Future research should consider an exploration of the most important influencers in changing perceptions on gender roles in parenting among community members. In addition, the role of female caregivers precluding the involvement of fathers should be investigated.

## Data Availability

The datasets used and/or analysed during the current study are available from the corresponding author on reasonable request.

## Abbreviations

ACK: Anglican church of Kenya
ADS-Nyanza: ACK development services of Nyanza
APHRC: African population and health research centre
ANC: Antenatal care
ECD: Early childhood development
ESRC: Ethics and scientific review committee
FGD: Focused group discussion
HIV/AIDS: Human immunodeficiency virus/acquired immune deficiency syndrome
IDI: In-depth interview
IRB: Institutional review board
KAP: Knowledge, attitudes, and practices
KCSE: Kenya certificate of secondary education
KII: Key informant interview
LMICs: Low- and middle-income countries
MCH: Maternal and child health
MOH: Ministry of health
NGO: Non-governmental organization
SSA: Sub-Saharan Africa
